# Yeast Species Associated with Industrial Cultures of the Marine Microalgae *Tisochrysis lutea*: Temperature Profiles and Auxin Production

**DOI:** 10.3390/jof11110818

**Published:** 2025-11-18

**Authors:** Madalena Matos, Mónica A. Fernandes, Natacha Coelho, Tamára F. Santos, João Varela, Alexandre M. C. Rodrigues, Isabel Sá-Correia

**Affiliations:** 1 iBB—Institute for Bioengineering and Biosciences, Instituto Superior Técnico, Universidade de Lisboa, Av. Rovisco Pais, 1, 1049-001 Lisbon, Portugal; madalena.matos@tecnico.ulisboa.pt (M.M.); monica.a.fernandes@tecnico.ulisboa.pt (M.A.F.); 2 Associate Laboratory i4HB—Institute for Health and Bioeconomy, Instituto Superior Técnico, Universidade de Lisboa, Av. Rovisco Pais, 1, 1049-001 Lisbon, Portugal; 3 Necton S.A., Belamandil, 8700-152 Olhão, Portugal; natacha.coelho@necton.pt (N.C.); alexandre.rodrigues@necton.pt (A.M.C.R.); 4 MED—Instituto Mediterrâneo para a Agricultura, Ambiente e Desenvolvimento, CHANGE—Global Change and Sustainability Institute, Faculdade de Ciências e Tecnologia, Campus de Gambelas, Universidade do Algarve, Ed. 8, 8005-139 Faro, Portugal; 5 Centre of Marine Sciences, Campus Gambelas, University of Algarve, 8005-139 Faro, Portugal; tfsantos@ualg.pt (T.F.S.); jvarela@ualg.pt (J.V.); 6 GreenCoLab—Associação Oceano Verde, Campus Gambelas, University of Algarve, 8005-139 Faro, Portugal; 7 Department of Bioengineering, Instituto Superior Técnico, Universidade de Lisboa, Av. Rovisco Pais, 1, 1049-001 Lisbon, Portugal

**Keywords:** yeast species, yeast isolation, marine yeasts, *Rhodotorula*, industrial microalgae cultivation, yeast-microalgae association, temperature profiles, auxin production

## Abstract

This study provides the first systematic characterization of culturable yeast diversity associated with large-scale cultivation of *Tisochrysis lutea*. This marine haptophyte is widely used in aquaculture for its high content of essential fatty acids, pigments, and other bioactive compounds. Culture sampling was conducted at Necton S.A. facilities (Olhão, Portugal) over full production cycles from 5 L flasks until tubular photobioreactors during the months of May and June. The study aimed to identify and isolate the present yeast species and evaluate their physiological traits relevant to potential co-cultivation strategies. All retained isolates belonged to the phylum Basidiomycota, with six species identified: *Rhodotorula sphaerocarpa* (45%), *R. mucilaginosa* (20%), *R. diobovata* (13%), *Vishniacozyma carnescens* (16%), *Naganishia diffluens* (3%), and *Moesziomyces aphidis* (3%). Temperature growth profiles (10–40 °C), tolerance to artificial sea water, and auxin production were characterized, revealing that, except for *V. carnescens*, the yeast isolates grow optimally at 25–30 °C, within the ideal range for *T. lutea* cultivation. Results suggest that some of these marine yeasts, particularly *R. sphaerocarpa* and *R. mucilaginosa* isolates, could serve as biological enhancers of algal productivity, in situ. This foundational work supports future efforts to develop targeted yeast management or co-cultivation strategies, with the goal of improving biomass yield and metabolite production in industrial *T. lutea* photobioreactors.

## 1. Introduction

The increasing global demand for sustainable bioresources has intensified interest in microalgae as versatile platforms for producing high-value compounds, including lipids, pigments, biofuels, polysaccharides, nutraceuticals, and other bioactive metabolites [1,2,3]. For this reason, the cultivation of microalgae has gained increasing attention in industrial biotechnology [3]. Among marine microalgae, *Tisochrysis lutea*, formerly known as *Isochrysis galbana* (Tahiti isolate)—a haptophyte known for its high docosahexaenoic acid (DHA) content and fast growth rate—has emerged as a promising candidate for industrial-scale aquaculture and biotechnological applications [2,4,5]. *T. lutea* is widely used in hatcheries for larval nutrition, most commonly to feed oyster and shrimp larvae and has gained attention for potential applications in functional foods and nutraceuticals due to its rich composition of omega-3 fatty acids, sterols, and carotenoids such as fucoxanthin [2,6,7]. The species name “*lutea*” stems from its saffron yellow color, being a golden single-cell algae [7].

In natural and engineered environments, microalgae coexist with complex microbial communities, including bacteria and fungi, that, together, form the algal microbiome. These microbial consortia can significantly influence the growth, productivity, and metabolic activity of the algal host [8,9,10,11]. Among the various microorganisms associated with industrial microalgal cultures, although present at low concentrations, yeasts represent a particularly interesting group [12]. These unicellular fungi can engage in mutualistic, commensal, or competitive interactions with microalgae, depending on environmental conditions and species involved [13]. In some cases, yeasts enhance algal productivity by providing essential growth factors, such as vitamins, or by modulating the microenvironment through the production of signaling molecules and enzymes [14,15]. In other scenarios, yeasts may act as contaminants that compete for nutrients, secrete inhibitory metabolites, or alter the physicochemical properties of the culture medium [16]. Understanding the role of yeasts is crucial for optimizing industrial-scale cultivation systems. Through microbiome engineering, co-cultivation strategies, or targeted microbial management, it may be possible to harness beneficial yeast–microalgae interactions while minimizing negative impacts [9,17,18]. This knowledge can lead to more stable, resilient, and productive algal bioprocesses.

This study is dedicated to the monitoring of yeast culturable diversity associated with a large-scale cultivation of *Tisochrysis lutea* at Necton S.A. facilities, Portugal, from 5 L flasks until tubular photobioreactors (max. 18,000 L), during the months of May and June of 2024. A previous study also envisaging the isolation of the culturable yeast population in the same industrial setting, but in association with *Microchloropsis gaditana* cultivated from 5 L to 27,000 L, was carried out during the months of February to May of 2024 [12]. This first report revealed a high percentage of red yeasts (90% of the total yeast isolates retained), with the capacity to produce lipids and biosurfactants and suggested their potential use as probiotics in industrial microalgae production [12]. Since the yeast populations isolated from microalgal cultures and from water samples from Ria Formosa, that are used to fill the bioreactors after disinfection, share yeast species of phylogenetically close isolates, this common source was suggested [12].

Given that there are culture conditions influencing the structure of the associated microbial communities, such as light intensity, temperature, salinity, pH, nutrient availability, the temperature profiles, and the effect of synthetic sea water on the growth of independent isolates belonging to the different yeast species identified were examined under standardized laboratory conditions [3,19,20]. Microbial communities in the phycosphere (the microenvironment surrounding the algal cells) are known to fluctuate with temperature changes, which directly affect their functionality [3,20,21]. The overall growth rate, pigment composition, and nutrient uptake of microalgae could be directly impacted by these shifts, emphasizing the need for careful temperature regulation in cultivation systems [22,23]. Despite the commercial relevance of *T. lutea*, large-scale cultivation is still constrained by environmental and physiological factors that limit biomass yield and biochemical productivity [24]. In this context, the use of phytohormones, such as indole-3-acetic acid (IAA), a naturally occurring auxin, has gained interest as a strategy to enhance microalgal growth and metabolism [25]. Auxins, particularly IAA, are known to regulate key processes such as cell division, elongation, and differentiation in higher plants, and emerging evidence suggests similar growth-promoting roles in microalgae [26]. Recent studies have demonstrated that exogenous IAA can stimulate photosynthesis, pigment biosynthesis, lipid accumulation, and stress tolerance in both freshwater and marine algal species [27,28]. In microalgae, low concentrations of auxins can dramatically stimulate biomass accumulation, lipid synthesis, pigment production, and enhance resistance to abiotic stressors such as heavy metals or nutrient deficiency [29,30]. However, the effects of auxins are complex and dose dependent; excess IAA may lead to oxidative stress or growth inhibition, while suboptimal levels may fail to elicit significant physiological responses [28]. Unlike in vascular plants, where auxin biosynthesis and signaling are well-characterized, the physiological roles of auxins in microalgae remain less understood [26]. However, both endogenous IAA production and exogenous auxin sensitivity have been documented in multiple algal taxa [31]. Yeasts, including both plant-associated and endophytic strains, can produce IAA under specific culture conditions. Several yeast species have been shown to produce IAA in vitro, with concentrations ranging from tens to hundreds of µg/mL, depending on species and fermentation conditions [32,33]. Yeasts capable of auxin production present a promising yet underutilized avenue for enhancing microalgal growth and metabolite production through natural, biologically mediated hormone delivery. This study is dedicated to the isolation and identification of yeast culturable diversity associated with industrial cultures of *T. lutea* and to the characterization of the temperature profiles, effects of artificial sea water on growth, and to auxin production by isolates of different species. The phylogenetic analysis, as well as the characterization of the temperature profiles and effects of artificial sea water, was also performed for isolates obtained in association with *M. gaditana*, cultivated in the same industrial setting. This study is a first step towards co-cultivation strategies or targeted microbial management to harness beneficial yeast–algal interactions.

## 2. Materials and Methods

### 2.1. Tisochrysis lutea Culture Sampling for Yeast Isolation

**Figure 1:**
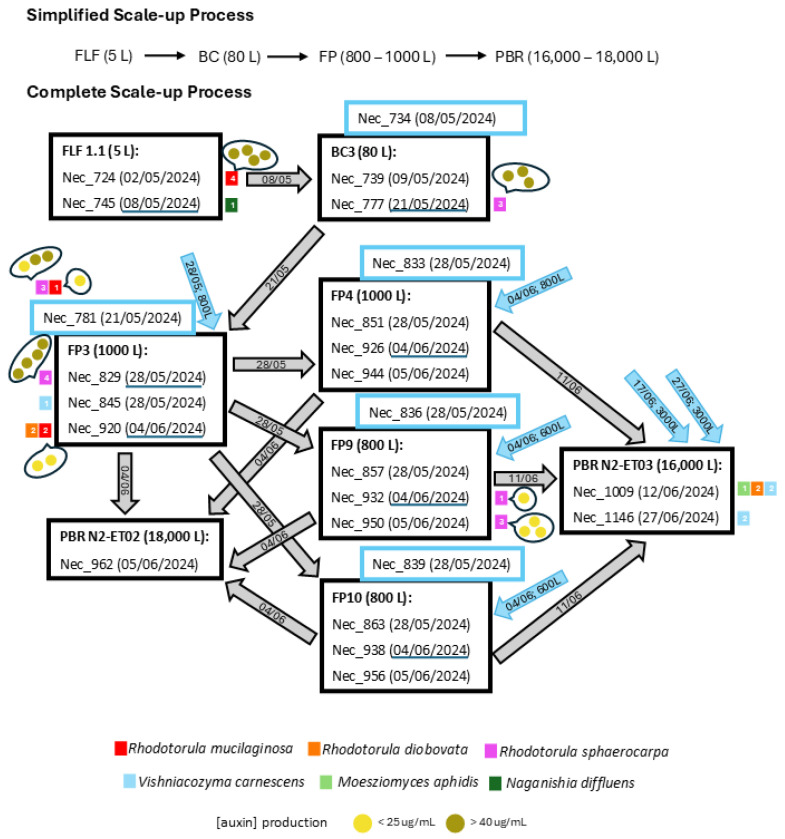
Schematic representation of the microalgae *Tisochrysis lutea* production flow. The samples analyzed, named as Nec_NNN, in which NNN is the sample number, are indicated together with the collection date, within brackets. Water samples are indicated inside blue squares. The reactor type and the volumetry are in bold. The small colored squares next to each analyzed sample indicate the species (differentiated by color) isolated from the specific sample; inside the square is the number of isolates retained from each sample. The dates of the passages between the reactors are underlined in dark blue. Blue arrows indicate the volume of water added to the reactor and the addition date. Circles represent the level of auxin production by *R. mucilaginosa* and *R. sphaerocarpa* isolates. FLF—5 L Flat Bottom Flasks, BC—Bubble Column, FP—Flat Panel, PBR—Photobioreactor.

Sampling for yeast isolation was performed during the scaling-up of the industrial cultivation of *Tisochrysis lutea* at Necton S. A. facilities (Olhão, Portugal), and this is schematized in Figure 1. The strain was kindly provided by Wageningem University. The microalgae cultivation started in the laboratory by a pre-culture with a concentration of approximately 0.4 g/L dry weight, growing in NutriBloom^®^Plus medium in 5 L Flat Bottom Flasks (FLF). The FLF were aerated (with intermittent CO_2_ injections) and exposed to 24 h LED lighting. These FLFs were used to inoculate aerated 80 L Bubble Columns (BC) with controlled CO_2_ injection and 24 h LED lighting. BCs were, in turn, used to inoculate outdoor Flat Panels (FP)-plastic bags of 800 L or 1000 L (with 0.08 m or 0.1 m width, respectively, 10 m wide, and 1 m of water column) supported by a metal structure, with filtered aeration and controlled CO_2_ injection. Finally, several FPs are used to inoculate 16,000 L or 18,000 L tubular photobioreactors (PBR).

Aliquots of 50 mL culture were collected before and after the passage to new bioreactors. In the PBRs, samples were collected every week. When possible, before passage to a new reactor, disinfected sea water samples were also collected. The sea water used for microalgae cultivation was collected from Ria Formosa Natural Park (Olhão, Portugal), decanted, ultrafiltered at 0.02 µm, chlorinated, neutralized with thiosulfate, and finally treated with UV (except in FLF, where autoclaved sea water was used). After these treatments, the water was pumped from the water deposit, through pipes, to the BCs, FPs, and PBRs, where the water sampling took place. The water was then supplemented with the culture medium NutriBloom Plus^®^ (Necton, Portugal), prior to culture inoculation. A total of 24 samples were processed for yeast isolation: 19 *T. lutea* culture samples and five water samples collected from 2nd May 2024 to 27th June 2024.

### 2.2. Isolation of T. lutea-Associated Yeasts

For yeast isolation, the collected samples of microalgal suspensions were serially diluted (10-fold) in 50% (*v*/*v*) Artificial Sea Water [ASW; 23.38 g/L NaCl (PanReact, Barcelona, Spain), 2.41 g/L MgSO_4_·7H_2_O (LabChem, Zelienople, PA, USA), 1.9 g/L MgCl_2_·6H_2_O (Fluka Analytical, Buchs, Switzerland), 1.11 g/L CaCl_2_·2H_2_O (Merck, Darmstadt, Germany), 0.75 g/L KCl (Merck), 0.17 g/L NaHCO_3_ (LabChem) and ddH_2_O]. The original samples and, when adequate, diluted samples, were spread (100 µL) onto YPD-Chl-agar [10 g/L yeast extract (VWR Chemicals, Radnor, PA, USA), 20 g/L peptone (BD Gibco, Waltham, MA, USA), 20 g/L glucose (Scharau, Barcelona, Spain), 20 g/L agar (LabChem), and 50% (*v*/*v*) ASW] plates supplemented with 100 µg/mL chloramphenicol (Chl; Sigma, Burlington, MA, USA).

Because of the expected low yeast concentration, whenever necessary, 12 mL of the initial microalgal suspensions were centrifuged at 13,000× *g* for 10 min, and the pellet resuspended in 400 µL 50% (*v*/*v*) ASW and the concentrated samples were plated onto YPD-Chl-agar plates, as previously described. Additionally, an enrichment culture was prepared using liquid YPD-Chl medium (composition as YPD-Chl-agar medium without agar) with the original sample constituting 10% (*v*/*v*) of the final volume. The culture was incubated at 22 °C with orbital agitation for up to 48 h. Every 24 h, 10-fold dilutions were prepared, and the culture and the diluted cultures were plated onto YPD-Chl-agar, as described.

All the plates were incubated at 22 °C for up to 10 days. Following incubation, the plates were observed, and the colonies were counted. Colonies of different morphologies, if present, were selected, and cells were observed on an Axioplan microscope (1000×) (Zeiss, Oberkochen, Germany) to differentiate yeast colonies from colonies of chloramphenicol-resistant bacteria. Isolated colonies (two to four colonies with different morphologies, whenever possible) were picked and streaked for purity onto the YPD-Chl-agar medium. For long-term storage, yeast isolates are preserved at −80 °C in their isolation medium containing 15% (*v*/*v*) glycerol included in the IST-Yeast CC (Instituto Superior Técnico Culture Collection, Lisbon, Portugal; https://blueyeastscc.tecnico.ulisboa.pt/).

### 2.3. Molecular Identification of Yeast Isolates

For molecular identification of yeast isolates, genomic DNA was extracted with the method described by Lee et al. (2012) [34] and used as a template for amplification by Polymerase Chain Reaction (PCR) of the D1/D2 domain sequence of the 28S ribosomal DNA (rDNA) and the internal transcribed spacer (ITS) region of rDNA. The primer pairs used for D1/D2 and ITS amplification were, respectively, NL-1 (5′-GCATATCAATAAGCGGAGGAAAAG-3′) and NL-4 (5′-GGTCCGTGTTTCAAGACGG-3′), and ITS1 (5′-TCCGTAGGTGAACCTGCGG-3′) and ITS4 (5′-TCCTCCGCTTATTGATATGC-3′), which are considered effective for the taxonomic identification of yeasts [35]. The PCR protocol included a denaturation step at 98 °C for 30 s, followed by 35 cycles of denaturation at 98 °C for 10 s, annealing at 52 °C for 20 s, and extension at 72 °C for 30 s. A final extension was performed at 72 °C for 10 min. To amplify the DNA fragments, the Phusion™ High-Fidelity DNA Polymerase (Thermofisher Scientific, Waltham, MA, USA) was used. After amplification, the two amplicons for each isolate were purified using the NZYGelpure kit (NZYTech, Lisbon, Portugal) and Sanger-sequenced. The yeast species were identified by running a nucleotide BLAST (http://www.ncbi.nlm.nih.gov/blast, assessed on 12 September 2024) with the D1/D2 and ITS-obtained sequences against the core nucleotide NCBI database and all the D1/D2 and ITS sequences were submitted to GenBank.

### 2.4. Phylogenetic Analysis of Yeast Isolates from T. lutea and M. gaditana Cultures

For the phylogenetic placement of 75 of the 178 yeast isolates obtained from *T. lutea* cultivation (32 isolates described in this work) and *M. gaditana* large-scale microalgae culture performed before in the same industrial setting (146 isolates) [12], corresponding to one isolate per species obtained per microalgal culture sample [Supplementary Material Table S1 (isolates from culture samples) and Table S2 (isolates from water samples), bold], the D1/D2 consensus rDNA sequences were aligned iteratively with the sequences of the type strains for each species, whose GenBank accession numbers are given on Supplementary Material Table S3. Multiple alignment was carried out with Muscle, available in the software MEGA-X v.11. The Mega-X software was also used for the phylogenetic tree construction using the maximum likelihood method of the Kimura 2-parameter model, selected based on the MEGA-X software recommendation considering the data [36,37]. The confidence level of the clades was estimated using a bootstrap analysis with 500 replicates.

### 2.5. Temperature Profiles of Isolates Belonging to the Eight Yeast Species Isolated from T. lutea and M. gaditana

Amongst the 178 yeast isolates obtained from *T. lutea* (32 isolates described in this work) and *M. gaditana* cultivations (146 isolates) [12], 15 isolates were selected (Table S4) to obtain the temperature profiles for the eight different species [38]. Whenever available, two isolates of each species from different microalgal cultures were selected for testing.

Yeast cells were pre-cultured in liquid YPD medium (prepared with 50% (*v*/*v*) ASW) for 24 h with orbital agitation and incubated at 22 °C. Pre-cultured cells were harvested by centrifugation at 4600× *g* for 5 min at 4 °C and inoculated in 20 mL of minimal medium [6.7 g/L of Yeast Nitrogen Base (BD Difco), 20 g/L of glucose, and ddH_2_O with pH adjusted to 5.5] in 100 mL shake flasks, at an initial optical density at 600 nm (OD600nm) of 1. Additionally, a culture medium with 100% ASW was also used for the inoculation of the pre-cultured cells. The media were filter sterilized using a 0.2 µm filter (Whatman^®^ Puradisc, Maidstone, UK). The cultures were incubated at different temperatures (10, 15, 20, 25, 30, 35, 40 °C) with orbital agitation, and growth was monitored by measuring OD600nm using a U-2000 HITACHI spectrophotometer (Tokyo, Japan).

The maximum specific growth rates were calculated by taking the slope of a linear regression from a semi-logarithmic plot of optical density versus time, focusing specifically on the exponential growth phase for each isolate at each temperature tested.

### 2.6. Assessment of Auxin Production Using the Salkowski Reagent

The 32 yeast isolates obtained from *T. lutea* cultures were tested for auxin production capacity. To quantify auxin production, yeast cells were pre-cultured in liquid YPD medium for 24 h with orbital agitation and incubated at 22 °C (isolation temperature). Pre-cultured cells were harvested by centrifugation at 4600× *g* for 5 min at 4 °C and inoculated in 20 mL of minimal medium in 100 mL shake flasks, at an initial optical density at 600 nm (OD600nm) of 1. The cultures were incubated at 22 °C with orbital agitation, and growth was monitored by measuring OD600nm using a U-2000 HITACHI spectrophotometer. This medium was filter sterilized using a 0.2 µm filter (Whatman^®^ Puradisc).

The assessment of auxin production under standardized conditions was carried out based on the method with the Salkowski reagent, performed as described before [39,40]. Briefly, after 72 h of cultivation in the minimal medium, 500 µL of supernatant were mixed with 500 µL of Salkowski reagent [2 mL of 0.5 M FeCl_3_ (ThermoFisher) and 98 mL of 35% HClO_4_ (Merck)], and the intensity of the pink color of the mixture, after 30 min of incubation in the dark at room temperature, was quantified with a spectrophotometer at 530 nm. A calibration curve was made and prepared using indole-3-acetic acid (Sigma) as standard.

## 3. Results

### 3.1. Culturable Yeasts Obtained During the Upscaling of Tisochrysis lutea Industrial Cultivation

To characterize the culturable yeast populations present in the large-scale microalgal culture of *Tisochrysis lutea* produced and collected at Necton S.A. facilities, the scale-up process described in the Section 2.1 and schematized in Figure 1 was followed for two months (May to June 2024).

A total of 24 samples (19 microalgal culture samples and five samples of the disinfected water used for cultivation and sampled from the filled bioreactor) were received in the iBB-IST Laboratory for yeast isolation. The isolation was achieved following a protocol previously optimized by our team [12]. Shortly, YPD-Chl-agar plates (chloramphenicol-Chl supplementation was used to avoid susceptible bacterial growth) were inoculated either with the original sample, the concentrated sample, or with cultures resulting from enrichment of the original sample. Two to four colonies of different morphology present in the plates were selected and streaked onto YPD-Chl-agar plates. All the selected colonies were confirmed to be yeasts by microscopic observation. Chloramphenicol-resistant bacteria and filamentous fungi were also present on agar plates, at different concentrations depending on the inoculated samples. In some cases, filamentous fungi growth on the isolation plates prevented yeast isolation.

**Figure 2:**
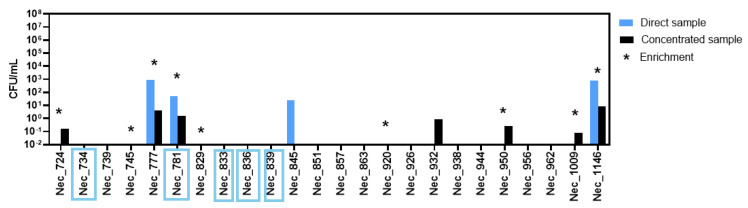
Concentration of culturable yeast cells in *Tisochrysis lutea* culture samples. Yeast concentration, as colony forming units (CFU) per milliliter of the original sample, was obtained for each microalgal culture sample and for each water sample. CFUs/mL were calculated by counting yeast colonies present onto YPD-Chl-agar plates inoculated with the original sample (direct sample, blue) or the concentrated sample (concentrated sample, black). The stars indicate the samples for which yeast colonies were obtained following an enrichment culture step. Water samples are those indicated inside blue squares.

Yeast abundance in *T. lutea* samples (Figure 2) was assessed in terms of colony forming units per milliliter of original samples (CFU/mL) on agar plates inoculated with the original samples or the concentrated original samples. When yeast isolates resulted from inoculation with the enrichment culture, an asterisk is used in Figure 2 to inform that yeasts were isolated given that their concentration in the original sample cannot be estimated.

Nineteen culture samples were collected during *T. lutea* cultivation scale-up. From nine of these samples, no yeast isolates were obtained. In samples Nec_777 and Nec_1146, yeast colonies were obtained in all the plating conditions (direct, concentrated, and enriched). From the remaining samples, yeast colonies were obtained only in one of the three conditions, with the exception of samples Nec_724, Nec_950, and Nec_1009, from which yeast colonies were isolated from both the concentrated and the enriched cultures. No yeast colonies were obtained from four of the five water samples analyzed. Remarkably, the CFU/mL values of the concentrated condition are, in general, lower than those calculated from plating the original sample. This discrepancy was due to the higher concentration of filamentous fungi present in the concentrated sample which hindered yeast growth and/or yeast observation and isolation.

The processing of the 24 samples resulted in the retention of 32 yeast isolates, selected to obtain the maximum phenotypic variety (Tables S1 and S2).

### 3.2. Taxonomic Profiling of the Yeast Isolates

The 32 yeast isolates retained were molecularly identified based on the comparison of their D1/D2 and ITS nucleotide sequences with the sequences deposited in the NCBI database and their nucleotide sequences were submitted to GenBank (the accession numbers and isolation source are displayed in Supplementary Material Tables S1 and S2). All the isolates identified belong to known species.

**Figure 3:**
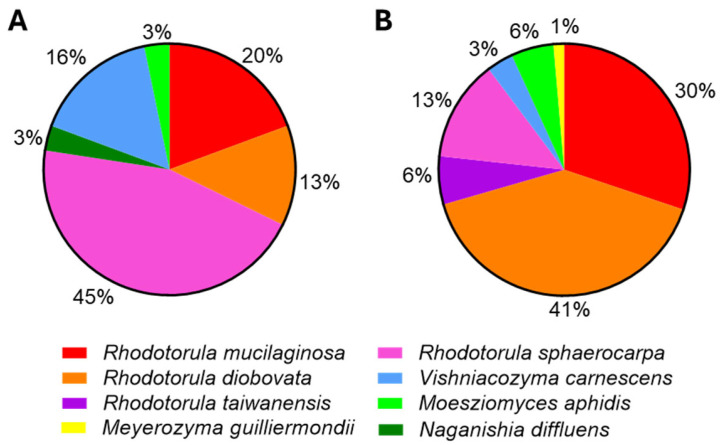
Distribution of the isolates obtained from the different yeast species identified. Yeasts were isolated from 24 samples collected during the upscale of *Tisochrysis lutea* industrial cultivation (this work) (**A**), and from 40 samples collected during the upscale of the *Microchloropsis gaditana* culture (**B**); this figure was prepared based on the results published by [12].

All the retained isolates belong to the phylum Basidiomycota, including three species of the oleaginous genus *Rhodotorula*, *R. sphaerocarpa* (45%), *R. mucilaginosa* (20%), and *R. diobovata* (13%), as well as *Vishniacozyma carnescens* (16%),* Naganishia diffluens* (3%), and *Moesziomyces aphidis* (3%) (Figure 3A). Most of the identified species were also found in a previous study regarding the scale-up cultivation of *M. gaditana* [12] (Figure 3B) although the percentage of isolated individuals per species varied. The variation observed in Figure 3 is not surprising considering the complexity of the microalgal scale-up process, with multiple entries of disinfected water and pre-cultures (Figure 1), the different number of analyzed samples and isolates obtained, and the differences in the temperature and other variables depending on the cultivation periods of the year, including the water used with origin in the Ria Formosa, followed by a disinfection process.

**Figure 4:**
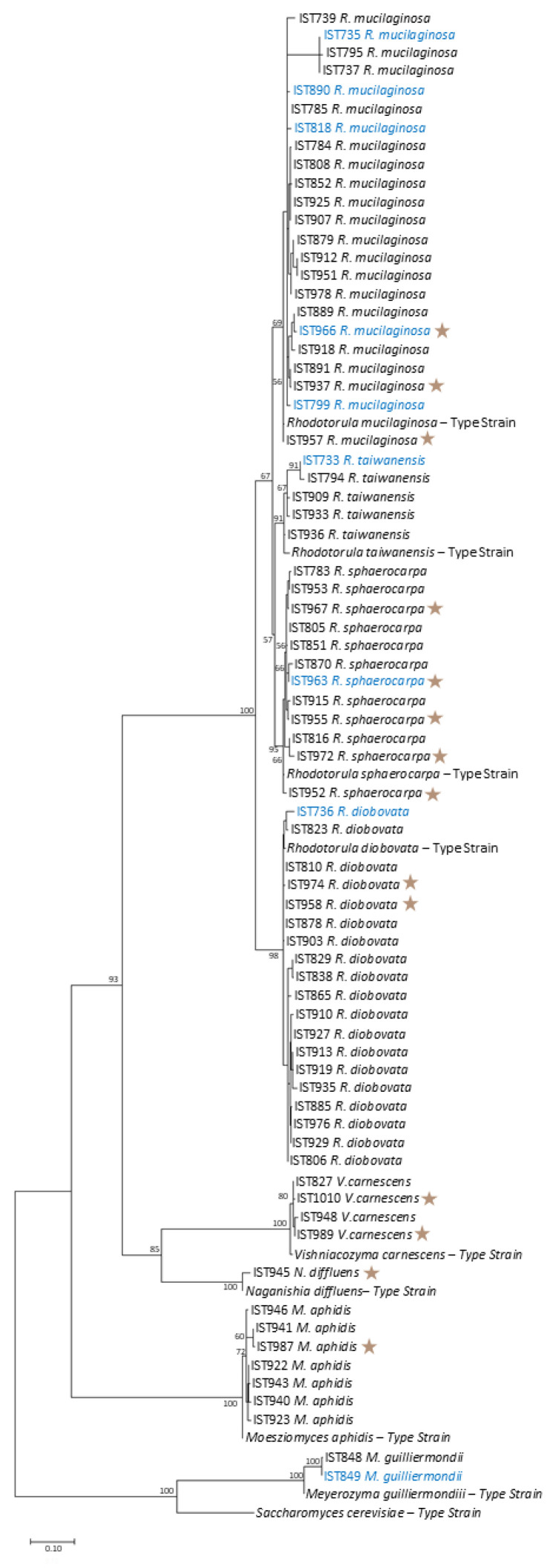
Phylogenetic analysis of yeast isolates (one isolate per species per sample) obtained from *T. lutea* (brown stars; this study) and *M. gaditana* [12] industrial cultivations at the same Necton S.A. facilities. The phylogenetic analysis was based on the alignment of sequences of the D1/D2 domain of the 28S rDNA region, inferred by means of the maximum likelihood method and Kimura 2-parameter model. Sequences from the type strains of the different yeast species were included. Isolates obtained from water samples are indicated in blue. The scale bar indicates the number of expected substitutions per site. The numbers provided at the branches are the frequencies (in percentage) of appearance of a given branch in 500 bootstrap replications.

The molecular identification, at the species level, of the isolates obtained from *T. lutea* cultures is consistent with the phylogenetic analysis performed, using D1/D2 consensus region and the maximum likelihood inference method, which placed the isolates-selected one per species, per sample-close to the type strains for each species (Figure 4). Moreover, these isolates (brown stars) have D1/D2 regions phylogenetically similar to isolates of the same yeast species isolated from *M. gaditana* cultures [12] and there is no clear phylogenetic difference between isolates obtained from water samples or culture samples. Nevertheless, only a small and highly conserved region of the genome is considered in Figure 4.

Notably, yeast isolates were obtained from both samples (Nec_724 and Nec_745) collected during the FLF stage, as indicated in Figure 1. The cultivation medium utilized in this step of the scale-up was autoclaved, which indicates that those yeast isolates from the *R. mucilaginosa* and *Naganishia diffluens* species might be already present in the microalgae stock culture. This phenomenon was also reported before [12]. Moreover, yeasts were also isolated from the water obtained from Ria Formosa, following a disinfection process and harvesting from the filled bioreactors. It is possible that these water samples have been contaminated in the reactor system after the ultrafiltration step.

### 3.3. Temperature Profiles of the Different Yeast Species Isolated from Microalgae Cultures

**Figure 5:**
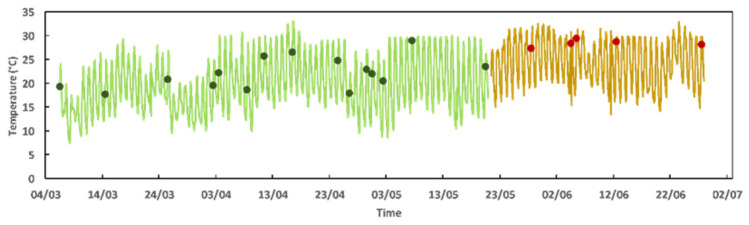
Variation of the temperature measured inside the Flat Panels and Photobioreactors during the scale-up of *M. gaditana* (green) and *T. lutea* (gold). The dots represent the sampling points-*M. gaditana* (green) and *T. lutea* (red)-as indicated in Figure 1. The temperature given represents an average of the temperature measured hourly in the reactors in use with the cultures.

**Figure 6:**
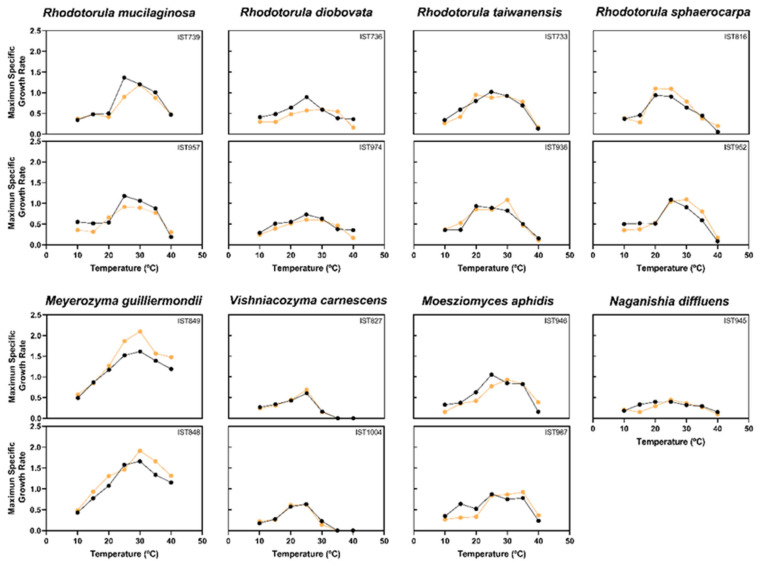
Temperature profiles of isolates belonging to the eight yeast species found in association with the two microalgae cultures during the scale-up process. The temperature profiles were obtained by yeast cultivation at temperatures in the range 10–40 °C in minimal medium (MM) supplemented (yellow) or non-supplemented (black) with 100% Artificial Sea Water (ASW). Maximum specific growth rates were calculated as described in the Section 2. The tested isolates are indicated in the corresponding panels.

The temperature profiles of the different yeast species isolated from *T. lutea* and *M. gaditana* [12] industrial cultivations were established using, whenever possible, two different isolates of each yeast species selected at random (Table S4). The two microalgal cultures were followed at distinct times of the year with subsequent temperature variations throughout. The maximum and minimum temperatures in the reactors of *T. lutea* and *M. gaditana* cultivation (Figure 5) were taken into consideration and a wide range of temperatures (from 10 °C to 40 °C) were tested (Figure 6). The 15 isolates selected at random were cultivated under standardized conditions in a defined minimal medium with glucose, adjusted to pH 5.5. To better understand the effect of sea water on growth at different temperatures, Artificial Sea Water (ASW) was used to supplement another set of growth experiments.

Within the experimental errors, the two tested isolates of the same species exhibit, in general, similar temperature profiles. In general, the optimum temperature for growth of the yeast species tested was between 25 °C and 30 °C but the isolates of *V. carnescens* revealed a lower optimum temperature between 20 °C and 25 °C and a maximum temperature for growth below 35 °C, which is a temperature that allows significant growth for all the other species, that can even tolerate 40 °C, in particular the species *M. guilliermondii*. Consistent with our results, *V. carnescens* was reported as psychrotolerant [41,42].

The addition of artificial sea water to the growth medium led to a consistent positive effect on the maximum specific growth rates for the two isolates tested of *M. guilliermondii* and of *R. sphaerocarpa* and to a slight negative effect on the growth of the two isolates tested of *R. mucilaginosa* and *R. diobovata*. No detectable effect was found for the other species, possibly due to their very low growth rate registered in the growth medium used.

### 3.4. Auxin Production by Yeast Isolates Obtained from T. lutea Cultures

**Figure 7:**
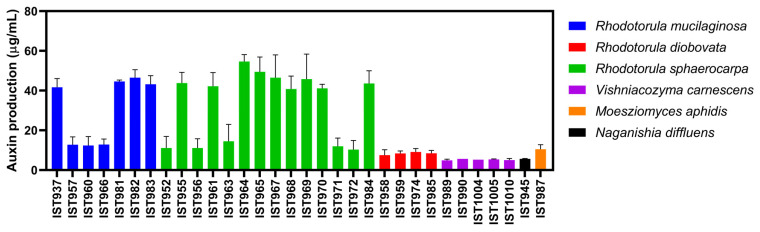
Auxin production of 32 yeast isolates belonging to the different species obtained from *T. lutea* industrial cultivation. Auxin production capacity was assessed after 72 h of cultivation under standardized growth conditions using the Salkowski reagent, as described in the Section 2. Data represents the average of three independent experiments, and the error bars indicate standard deviation.

The 32 yeast isolates obtained from *T. lutea* cultures were tested for their capacity to produce auxins after 72 h of growth under standardized conditions (Figure 7). The selection of this time-point was based on previous optimization experiments in the referred conditions.

Auxin production was found to be strain-dependent, as previously reported [40,43,44], with isolates of the same species being better producers than others. For all the isolates tested under standardized conditions, auxin production from *R. diobovata*, *Vishniacozyma carnescens*, *Moesziomyces aphidis* and *Naganishia diffluens* species was poor (between 4 and 15 µg/mL). This is particularly true for all five *V. carnescens* isolates and the sole *N. diffluens* isolate. However, since all the experiments were performed under standardized conditions, factors such as growth temperature and medium composition could have negatively impacted auxin production. Although exhibiting variable levels, *R. mucilaginosa* and *R. sphaerocarpa* isolates produce, in general, the highest concentrations of auxins (more than 40 µg/mL). Interestingly, all the *R. mucilaginosa* isolates producing the highest auxin levels (Figure 1) were isolated from the 5 L FLF stage of the scale-up (Nec_724), when microalgae cultures are kept under laboratory conditions and grown in a medium sterilized by autoclave. This may suggest a positive symbiotic interaction between high auxin producing isolates of *R. mucilaginosa* and the microalga.

## 4. Discussion

A promising approach for enhancing the industrially important *Tisochrysis lutea* biomass productivity and metabolite yield is microbial co-cultivation, specifically, the pairing of *T. lutea* with auxin-producing microorganisms. The yeast isolates capable of auxin production found in this work in association with *T. lutea* cultures may have a role in nutrient cycling and stress alleviation by producing antioxidants, and reveal a promising yet underutilized avenue for enhancing microalgal growth and metabolite production through natural, biologically mediated hormone delivery [11,30]. Although exogenous auxins or auxin-producing bacteria have been shown to stimulate growth and metabolite production in microalgae species [31,45], similar strategies have not yet been applied to *T. lutea*, nor has the potential of yeast-based auxin production been explored in this context. However, yeasts are generally non-pathogenic, easy to handle in industrial processes, and marine yeast species can produce significant levels of indole-3-acetic acid (IAA) under appropriate conditions [46]. The auxin-producing isolates associated with *T. lutea* industrial cultures gathered during this study, in particular some *R. mucilaginosa* and *R. sphaerocarpa* isolates, are expected to provide a sustainable, in situ source of auxins to promote algal growth [30]. Future controlled co-culture experiments are therefore required to assess their effects on *T. lutea* growth, lipid profile, and pigment accumulation, following the optimization of inoculation timing and yeast-to-algae biomass ratios, among other relevant conditions [47]. This future research should focus on exploring those biological resources as well as the results obtained during this work under phototrophic conditions relevant to industrial photobioreactors. If the integration of auxin-producing yeast isolates into *T. lutea* cultivation systems is successful, it could enhance process productivity and reduce production costs [46]. To guide the successful co-cultivation of auxin-producing yeasts in *T. lutea* large-scale cultures, it is instrumental the characterization of relevant physiological traits of yeast isolates of the different species found in association with two large-scale microalgae cultures in the same industrial setting (*T. lutea* and *Microchloropsis gaditana* cultures). This is the case of the characterization of the yeast’s temperature profiles between 10 °C and 40 °C, and of the effect of artificial sea water on growth in that temperature range.

Temperature and salinity are key environmental factors that may significantly influence the growth, metabolism, and interactions of marine microalgae and their associated yeasts [48,49,50]. Sea water salts, primarily composed of NaCl and trace minerals, impose osmotic stress that can alter cellular function, while temperatures in the range 10–40 °C modulate metabolic rates and stress responses [24,51]. Their effects are species-specific and context-dependent, influencing not only individual physiology but also the nature of interspecies interactions [52]. Marine yeasts exhibit halotolerance, often growing in up to 10% NaCl, though high salinity can suppress growth or alter metabolic output. *Rhodotorula* spp. are halotolerant, with some strains being capable of growing up to 10–16% NaCl [53]. In large-scale microalgae cultivation systems such as photobioreactors, where temperature control is often one of the most challenging operational factors, managing the temperature profile of the microbiota can have profound positive effects on productivity [19,50]. The large-scale *T. lutea* culture, which lasted for more than a month, mostly during the month of June, experienced very significant temperature fluctuations throughout the 24 h of the day, generally ranging from 15 to 32 °C. This meant that samples were generally collected from cultures at temperatures around 28 °C. However, the *M. gaditana* culture we monitored in a previous study [12] took place between March and mid-May, with temperatures generally fluctuating between 10 and 30 °C, which meant collecting samples at temperatures generally around 18 °C during the first, coldest month, and around 25 °C during the warmest months. Understanding the shifts in temperature- and salt-dependent-microbiota composition can help in designing regulated microbiomes that synergistically enhance algal yields, since the nature of these interactions is temperature- and salinity-dependent [50,54]. It must be considered that in co-cultivation systems, salt and temperature tolerance mismatch between microalgae and yeasts can lead to competitive imbalances [55]. For instance, yeasts may outcompete algae at lower salinities and higher temperatures, while algae may dominate in marine-like conditions. Optimal co-cultivation requires balancing salinity and temperature to support mutual or complementary growth, possibly through selection of robust, halotolerant strains [50,52]. Understanding these interactions is crucial for optimizing mixed cultures in aquaculture and bioproduct applications [56]. The growth and interactions of *T. lutea* and *Rhodotorula* spp., commonly found in marine environments, are strongly influenced by temperature and salinity. *T. lutea* generally thrives in moderate temperature ranges between 20 °C and 30 °C, where its photosynthetic activity and biomass productivity are (close to) optimal, while temperatures below 20 °C significantly reduce its growth rate, with substantial declines in biomass and metabolite production even at 15 °C [19,57]. *Rhodotorula* yeasts, including the species examined in this work, *R. mucilaginosa*, *R. diobovata*, *R. taiwanensis*, and *R. sphaerocarpa*, are mesophilic, showing optimal growth between 25 °C and 30 °C. Elevated temperatures beyond 35 °C can lead to oxidative stress, while lower temperatures slow metabolic activity. Except for *Vishniacozyma carnescens*, reported as psychrotolerant, with an optimal temperature in the range 20–25 °C, the other yeast species associated with the microalgal cultures are also mesophilic. Therefore, the yeasts isolated in this work, and those isolated before by Matos et al., 2025 [12], display optimal growth at temperatures suitable for co-culture in *T. lutea* bioreactors, aligning well with *T. lutea*’s preferred range. These yeast species also maintain metabolic activity at suboptimal temperatures, which is advantageous for maintaining consistent auxin production across temperature fluctuations commonly encountered in closed systems. Future studies should characterize their auxin production profiles under conditions closer to those used for the industrial cultivation of *T. lutea*, since it is essential to ensure that auxin synthesis remains active within the same thermal window as *T. lutea*’s optimal growth conditions. In industrial photobioreactors where thermal control is feasible, matching the temperature tolerance and performance profiles of both organisms is key to realizing the full potential of yeast–alga co-culture systems [50]. Yeast strains with overlapping thermal optima and stable auxin output at 22–28 °C are likely the most promising for scaling this biotechnological approach.

The explanation for the different percentages of yeast species found in association with the two microalgae cultures carried out during warmer or cooler periods of the year, namely the higher percentage of *R. sphaerocarpa* (43%) in *T. lutea* versus *R. diobovata* (41%) in *M. gaditana*, is not straightforward. Not only is the number of samples analyzed unequal, being lower for *T. lutea*, but they are different microalgae species which can also affect their microbiomes. Considering the temperature profiles of the different yeast species and the temperature changes that occurred, a significant alteration of the percentage of the different associated yeast species is not likely. However, at the higher temperatures occurring in the *T. lutea* reactors, the water entering the reactors can have slightly different salinities, especially in the summer, but salinity is only one parameter of water quality. A higher salinity of the water in the summer months could explain the higher percentage of *R. sphaerocarpa* associated with *T. lutea,* given the better growth of their isolates with sea water, while growth of the other *Rhodotorula* species, in particular *R. diobovata* and *R. mucilaginosa* isolates, is negatively affected by the presence of sea water. However, at Necton S.A. facilities, when salinity increases, the Ria Formosa water can be mixed before sterilization with freshwater from the borehole which has a high calcium content and, for this reason, this can also affect yeast growth.

The inclusion of yeasts in industrial microalgae cultivation systems offers numerous advantages [14]. By establishing beneficial microbe–algae interactions, yeasts can improve the sustainability of large-scale algal production while simultaneously enhancing the production of high-value compounds for aquaculture, nutraceutical, and biofuel industries [10,14,58]. As research continues to reveal the full scope of yeast–algae interactions, the optimization of these co-cultures holds great promise for the development of next-generation industrial microalgal processes.

## 5. Conclusions

This study presents the first systematic characterization of culturable yeasts associated with large-scale *Tisochrysis lutea* cultivation, identifying six Basidiomycota species, predominantly *Rhodotorula sphaerocarpa* and *R. mucilaginosa*. Most isolates exhibited optimal growth between 25 and 30 °C and tolerance to artificial sea water, conditions matching those of *T. lutea* photobioreactors. Several isolates, notably *R. sphaerocarpa* and *R. mucilaginosa*, produced auxins, indicating potential roles in promoting algal growth through natural hormone-mediated interactions. The alignment of physiological profiles between these yeasts and *T. lutea* suggests compatibility for co-cultivation under industrial conditions. Such interactions could enhance biomass productivity and metabolite yield while reducing process costs. Overall, these findings provide a foundation for developing targeted yeast management strategies to improve the efficiency and sustainability of *T. lutea*-based bioprocesses.

## Data Availability

The original contributions presented in this study are included in the article and Supplementary Material. Further inquiries can be directed to the corresponding author.

## Supplementary Materials

The following supporting information can be downloaded at https://www.mdpi.com/article/10.3390/jof11110818/s1, Table S1: Yeast isolates retained for further studies following isolation from the different samples of *Tisochrysis lutea* cultures schematically represented in Figure 1. Information on the isolate ID, isolation conditions, species, GenBank NCBI accession number of the D1/D2 and ITS consensus sequences deposited is provided. The isolates represented in bold are those selected for construction of a phylogenetic tree; Table S2: Yeast isolates retained for further studies following isolation from the different water samples used for *Tisochrysis lutea* cultivation. Information on the isolate ID, isolation conditions, species, GenBank NCBI accession number of the D1/D2 and ITS consensus sequences deposited is provided. The isolates represented in bold are those selected for construction of a phylogenetic tree; Table S3: GenBank NCBI accession number of the D1/D2 of the type strains of the different species isolated; Table S4: Yeast isolates selected for the temperature profiling. Information on the isolates ID, their microalgae culture and sample collection date are provided.
